# “Generalist” Aphid Parasitoids Behave as Specialists at the Agroecosystem Scale

**DOI:** 10.3390/insects11010006

**Published:** 2019-12-19

**Authors:** Stéphane A.P. Derocles, Yoann Navasse, Christelle Buchard, Manuel Plantegenest, Anne Le Ralec

**Affiliations:** 1 IGEPP, Agrocampus Ouest, INRA, Université de Rennes 1, Université Bretagne-Loire, 35000 Rennes, France; 2 IGEPP, Agrocampus Ouest, INRA, Université de Rennes 1, 35650 Le Rheu, France

**Keywords:** aphid, parasitoid, specialist, generalist, host range, agroecosystem, trophic interactions, cytochrome c oxidase I, maximum likelihood

## Abstract

The degree of trophic specialization of interacting organisms impacts on the structure of ecological networks and has consequences for the regulation of crop pests. However, it remains difficult to assess in the case of parasitoids. Host ranges are often established by listing host records from various years and geographic areas in the literature. Here, we compared the actual hosts exploited at a local farm-scale by aphid parasitoids (Hymenoptera: Aphidiinae), to the available species listed as hosts for each parasitoid species. We sampled aphids and their parasitoids in cultivated and uncultivated areas in an experimental farm from April to November 2014 and thereafter used DNA-based data to determine whether a differentiation in sequences existed. Twenty-nine parasitoid species were found on 47 potential aphid hosts. Our results showed that the great majority of the parasitoid tested used fewer host species than expected according to data published in the literature and parasitized a limited number of hosts even when other potential hosts were available in the environment. Moreover, individuals of the most generalist species differed in their DNA sequences, according to the aphid species and/or the host plant species. At a local scale, only obligate or facultative specialist aphid parasitoids were detected. Local specialization has to be considered when implementing the use of such parasitoids in pest regulation within agroecosystems.

## 1. Introduction

Trophic specialization has been extensively studied because it strongly influences the spatial distribution of species and the structure of ecological networks. However, the evolutionary processes affecting the degree of trophic specialization remain highly debated. According to Loxdale et al. [1], species evolution toward true trophic generalism is very unlikely, particularly because ecological specialization lowers the competition for resources and allows survival in new habitats or niches (see Rundel and Nosil [2] for a review of ecological specialization processes). Important support for Loxdale’s statement is the recent discovery revealed following DNA analysis that most so-called generalists actually consist of many cryptic species [3,4,5,6]. In opposition to this view, Dennis et al. [7] have argued that generalist species actually exist in nature and that trophic generalism has its own benefits. One of the main benefits is the reduction of the extinction risk. For example, this risk of extinction is significantly reduced if a species is able to maintain itself on several resources in an environment where exploitable resources are hardly available or already exploited by other competitors. Because of this, Dennis et al. [7] advocate a balance between specialist and generalist species and therefore for the existence of generalists in nature (a view supported by Clarke [8]).

To balance the benefits of generalism, a trade-off should exist between the ability of a species to exploit a wide range of resources and the performance in the exploitation of these resources [6,9,10,11]. Ecological specialization is the consequence of this trade-off. Ecological niches of specialized species are consequently restricted to a narrow range of environmental conditions and resources [12,13,14]. Specialist species develop physiological and behavioral adaptations precisely matching their preferred habitat and resource characteristics [15]. Such adaptations are under selective pressure because they induce an increase in the efficiency of the use of the optimal resource [9,16]. As a result, the performance of specialists is in theory higher than those of generalist species under conditions matching the ecological niche of specialists [10]. Generalists tend to be favored in an environment where resources are scarce, or in unstable environments, and are therefore not available for some specialist species [10,17].

Agroecosystems are typical examples of disturbed environments with a large instability in resource availability. Such instability of the environment and resources is expected to promote generalist strategies [18]. However, agroecosystems tend to consist of a mosaic of cultivated and uncultivated areas greatly differing in their level of instability. Thus, because of the instability induced by seasonal harvests, we expect to find generalist species in cultivated areas [19,20,21] while uncultivated habitats (e.g., herbaceous strips, hedgerows, or woods), which are much more stable and host a large diversity of plants and resources [22,23], are expected to promote specialists.

The degree of species specialization of parasitoids in agroecosystems is particularly important because it has major implications in the ecosystem services in terms of pest regulation provided by natural enemies. It has a direct impact on the efficiency of the pest control as some host species could be more exploited than others. It also impacts on the ability of the natural enemies to maintain themselves in the local environment in the absence of pests [24,25] by using non-pest species. At the community level, Raymond et al. [26] suggest that the highest biocontrol efficiency might be achieved by the co-occurrence of specialist and generalist parasitoid species. Despite the importance of this trait in the pest regulation services, the actual range of resources used by many natural enemies in the local environment is poorly known. This gap of knowledge is a consequence of problems of identification and quantification of trophic interactions in ecosystems [26,27,28,29,30,31]. Host–parasitoid trophic interactions are, however, easier to identify than predator–prey interactions as emerging parasitoids can be identified from parasitized hosts collected on plants and therefore trophic links can be directly identified [32].

As a consequence, host–parasitoid interactions are a good biological model to examine the specialist-generalist debate as well as a major model for the study of the ecosystem service of pest regulation. In particular, there exists an extensive literature on aphid–parasitoid interactions. In theory, a large range of levels of specialization exists among the four hundred species belonging to the Aphidiinae subfamily (Hymenoptera: Braconidae) [33]. Indeed, while most parasitoid species are able to parasitize only one or two aphid species, some species are known to be able to parasitize several dozen aphid species [34,35]. However, data on these interactions are mainly qualitative and arise from the merging of disparate observations collected at various geographical locations and time scales. This could lead to an overestimation of the actual host range of many if not most parasitoid species [36], especially for the so-called generalist species. Moreover, because of the qualitative nature of most of the data, it is impossible to conclude on the possible ecological role (i.e., a pool of resources limiting the risk of extinction when the main host is absent) played by the rarely exploited resources (i.e., aphids) in a local environment.

Consequently, it is not so surprising that recent studies have revealed intraspecific host specialization [37,38], host-based genetic structuration, or even cryptic species in some generalist Aphidiinae such as *Aphidius matricariae, Diaeretiella rapae*, *Praon volucre* [5,39], *Binodoxys communis* [40], *Lysiphlebus fabarum* [41], and *Ephedrus plagiator* [42]. These convergent results question the existence of truly generalist Aphidiinae (i.e., species with populations able to successfully parasitize many different host species) and tend to support the “improbability of generalism” assertion in nature [1]. Nevertheless, because these studies still test populations from putative generalist species sampled in several geographic locations (sometimes separated from hundreds of kilometers) and over a wide time-scale (sometimes several years), the question of the actual behavior of the most generalist parasitoid species at a local scale remains overlooked.

In the present study, our goal was to assess the actual host range (i.e., the degree of trophic specialization) of aphid parasitoids from the subfamily Aphidiinae (Hymenoptera: Braconidae) in cultivated and uncultivated habitats at the local farm scale. Overall, we wanted to investigate whether the species considered in the literature as generalist actually parasitize a wide range of aphid species at a local scale. For this, a large sampling of aphids and their associated Aphidiinae was conducted at a farm-scale from April to November 2014. We examined plant–aphid–parasitoid interactions to test whether:

1. A correlation exists between the host range described in the literature and the number of aphid species truly parasitized in the agroecosystem by each identified parasitoid species
2. Parasitizing a wide range of aphid species actually limits the local risk of extinction of the parasitoid species. As a consequence, the more aphid species a parasitoid species is able to parasitize the longer the parasitoid species remains in the environment with a high population size
3. The availability of resources (i.e., aphid abundances), the sampling season, the type of habitats (cultivated or uncultivated) have an impact on the observed host range of the sampled parasitoid species. We hypothesized that because of the unstable nature of cultivated areas, generalist species are more likely to be found in these types of habitat, while specialist species are rather found in uncultivated areas, which are known to be more stable.

Lastly, as a large number of studies have demonstrated the existence of host specialization (if not cryptic species as such), we used DNA-based data on the five parasitoid species collected on the highest number of aphid species to determine whether, even at a local geographical scale, host-associated differentiations in DNA sequences exist. For this, we relied on a maximum likelihood tree built from the sequencing of three different genes (the mitochondrial gene cytochrome oxidase I, the nuclear long-wavelength rhodopsin and the ribosomal gene 16s, as these had earlier been shown to be relevant in the detection of cryptic species [5]).

## 2. Materials and Methods

### 2.1. Experimental Site and Sampling Methods

**Figure 1:**
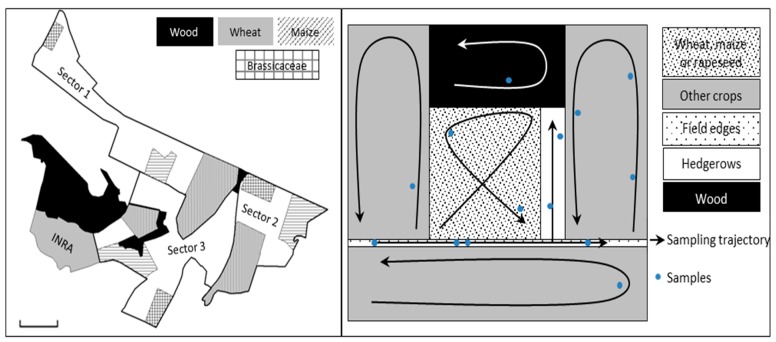
(**left**) Experimental area, block division, and field sampling; (**right**) example of sampling trajectory in crops and adjacent habitats. A sample includes up to five colonies of a unique aphid species sampled on the same plant in the same habitat at the same date.

This study was performed in the INRA experimental station of Le Rheu (Brittany, France) including 50 hectares of experimental fields, in an agricultural area of 9 km^2^ (UTM (zone 30U) **X**: 589341 **Y**: 5328111). This environment shows a large diversity of cultivated and uncultivated habitats. The experimental area was divided into 3 blocks. Within each block, three plots were targeted: one plot included a field of Brassicacae crop (2 *Brassica napus* and 1 *Brassica oleracea*), one a field of wheat, and one a field of maize (Figure 1). A plot comprised the cultivated field, the uncultivated habitats surrounding the field (e.g., hedgerows, herbaceous strips, woods, or fallow), and the adjacent cultivated fields. The adjacent fields sampled were sown with Fabaceae (*Medicago sativa*, *Pisum sativum,* and *Trifolium* sp.), Brassicaceae (*Raphanus sativus*), Poaceae (*Hordeum vulgarae*), and Solanaceae (*Solanum tuberosum*). The sampling of aphid colonies and Aphidiinae parasitoid mummies was carried out on one day per week for 3 weeks per month from April to November 2014. Consequently, 24 sampling sessions were performed in each block. The sampling method consisted of a visual search for 30 minutes in each crop, and between 30 minutes to 2 hours at the field edges, according to the area size, length, diversity and height of the vegetation (Figure 1).

A maximum of five aphid colonies of the same morphospecies on the same plant, in the same habitat (cultivated or uncultivated) and at the same date was collected. Aphids were counted and identified in the laboratory according to Blackman and Eastop [43] to confirm the morphospecies identified in the field. The plants harboring aphid colonies were identified to at least the family level in the laboratory using the flora of Blamey and Grey-Wilson [44]. Aphid mummies were placed in a climatic chamber at 20 ± 2.0 °C, 60% ± 10% relative humidity and a photoperiod of L16: D8 in a Petri dish. Emerging parasitoids and aphid species were identified in the laboratory using several relevant identification keys [43,45,46,47,48,49,50,51]. In this article, we consider aphids of the same species, collected on the same plant from a single type of habitat and on the same date as a sample.

### 2.2. DNA Sequencing of the Most Generalist Species

To detect host-associated subgroups and reveal potential cryptic species or parasitic specialization, DNA sequencing was performed on the five parasitoid species with the largest host ranges species observed in this study. For this, we extracted the DNA from up to three individual adult parasitoids per plant-aphid association identified throughout the sampling season for each of the parasitoid species selected following a non-invasive method [52]. The DNA was amplified with three markers: cytochrome c oxidase I (COI), 16S, and long wavelength rhodopsin (LWRh) (Table 1).

These markers seem to be the most relevant in the study of cryptic species complexes in Aphidiinae [5]. PCR amplifications were performed out following Derocles et al. [5]. PCR products (COI, 16S, and LWRh) were purified and both strands sequenced (Sanger technology; [57]).

Sequences were edited using Bioedit 7.2.5 [58] and aligned with MAFFT version 7.452 (default parameters [59]. Alignments were translated into amino acids using MEGA version X (version 10.0) [60] to detect frameshifts or stop codons indicating pseudogenes. For LWRh, the 5′ intron was removed from the analyses because of large divergences in sequence impeding sequence alignment [52]. Only the remaining 520 bp were used in the analysis. *Cotesia flavipes* (Hymenoptera: Microgastrinae) was used as an outgroup. Parasitoids belonging to another subfamily of Braconidae (in particular from the genus *Cotesia*) have been classically employed as outgroups in phylogenetic studies devoted to Aphidiinae [5,61,62]. The sequences of *C. flavipes* used were GQ853456 (COI), DQ538530 (16S) and DQ538703 (LWRH). To detect putative clades in the five parasitoid species, a Maximum Likelihood tree was built for the three gene fragments concatenated. We combined the three genes using SEQUENCEMATRIX [63]. From this, we then constructed the phylogeny using the ML tree MEGA X (version 10.0).

### 2.3. Statistical Analyses

Firstly, the effects of the sampling date (month of collection) and the type of habitat (cultivated *vs* uncultivated area) on the overall presence/absence of parasitism in the samples (*i.e.* at least one parasitoid mummy found in the sample) were analyzed with a generalized linear model (binomial family). Similarly, the effects of the sampling date and the type of habitats on the number of parasitoid mummies per sample were analyzed with a generalized linear model (negative binomial family). Post hoc pairwise comparisons were carried out with the function “esticon” of the “doBy” package [64].

Next, we pooled the data per parasitoid species to determine for each parasitoid species identified in our field sampling:

1. The realized host ranges: number of aphid species parasitized in the field for each parasitoid species.
2. The potential host ranges: number of aphid species found in our field samples considered as potential hosts for each parasitoid species according to the literature. These potential host ranges (based only on the aphids collected) differ from the theoretical host ranges (which consider all aphid–parasitoid interactions described in the literature). To construct these potential host ranges, we examined all the literature and considered all binary interactions between aphids and Aphidiinae observed in Europe [36] (the list of literature examined can be found in the Supplementary Material of Derocles et al. [36]). We added to this literature the comprehensive aphid–parasitoid interactions list of *D. rapae* [65].

Comparisons between realized host ranges and potential host ranges provide information on whether parasitoid species exploit the full range of hosts available and suitable (determined by the literature data) or rather focus on a narrow range of aphid species. To test whether the parasitoid considered by the literature as generalist exploits a high number of aphid species, we tested the correlation between the potential host range size and the realized host range size for each parasitoid species using the Spearman’s correlation test. To detect if the ability to parasitize a higher number of host species impacts on the presence of parasitoid in the field, we then test the effect of host ranges (realized and potential) of parasitoid species collected on three different parameters using generalized linear models. The presence of each parasitoid was characterized using: (1) the number of months of presence (Poisson family), (2) the number of samples where at least one parasitoid was found (negative binomial family), and (3) the total number of parasitoids collected (negative binomial family). In other words, we analyzed the effect of the host range sizes on the three parameters stated above.

Next, we determined the effect of the following environmental conditions on the degree of specialization (i.e., host range size) in parasitoid species:

1. The host availability for parasitoid measured by aphid abundance;
2. The period of sampling (before or after the harvest of the crop, referred further in the article as “sampling season”);
3. The place of sampling (cultivated or uncultivated area, referred further in the article as “type of habitat”).

We tested the effect of these factors together on the realized and potential host range of parasitoid species collected using generalized linear models (Gaussian family).

To assess to what extent the results of our study are influenced by taxonomic uncertainty, we again performed all the statistical analyses described above but using a new individual grouping based on the phylogenetic analysis originating from the DNA study. To this purpose, all clusters with a bootstrap value greater than 90 were considered as a putative distinct species. We then calculated the realized host ranges of putative parasitoid species.

## 3. Results

### 3.1. Sampling Data

Of 331 samples collected, 140 contained mummies (11 samples comprised only mummies). Samples were collected on 11 different plant species in the cultivated areas and on 41 different plant species in the uncultivated areas, belonging to 16 families. Overall, we sampled approximately 65,400 aphids (216.17 ± 360.36 aphids per sample) belonging to 47 different taxa (species or genus; Supplementary Material 1)). Sixteen aphid taxa were never found to be parasitized. Some aphids could not be identified to the species level, in particular in the genera *Aphis* and *Uroleucon*. Eight aphid species were sampled in cultivated areas and 46 species in uncultivated areas. From the 2,120 parasitoid mummies collected, 1,584 Aphidiinae emerged. We identified 29 species of Aphidiinae (Table 2; sampling data in Supplementary Material 2), few individuals of which could not be identified to the species level. The other mummies did not emerge or a hyperparasitoid emerged. Hyperparasitoids were excluded from this study.

### 3.2. Parasitism Rates

**Figure 2:**
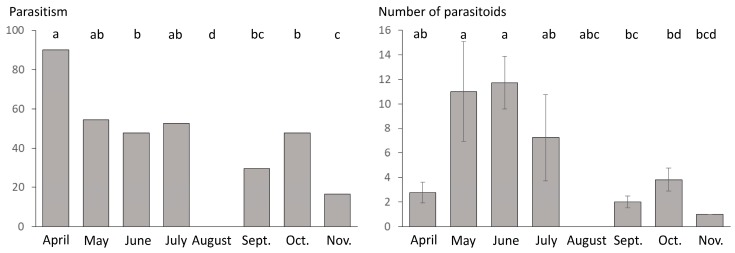
Percentage of samples where at least one parasitoid mummy was detected per sampling month (left) and the number of parasitoid mummies found per sample and per sampling month (right; mean ± standard error). Pairwise comparisons were carried out using “esticon” function as post hoc tests. Significant differences are indicated with different letters (*p* < 0.05).

Presence/absence of parasitism was significantly affected by the type of habitat (GLM; LR Chi-square = 19.061; df = 1; *p* < 0.001) and the sampling date (GLM; LR Chi-square = 20.918; df = 7; *p* = 0.004; Figure 2) but not by their interaction (GLM; LR Chi-square =2.83; df = 5; *p* = 0.726). Parasitoid mummies were found more frequently in the cultivated area (75.81% of the samples contained at least one mummy) than in uncultivated habitat (41.53% of the samples were parasitized). When testing the effect of the sampling date and the type of habitat on the number of parasitoid mummies found per sample, we detected only a significant effect of the sampling date (GLM; LR Chi-square = 31.841; df = 7; *p* < 0.001; Figure 2) and the type of habitat whilst the interaction of these two factors did not affect the number of mummies per sample (GLM; respectively LR Chi-square = 1.593; df = 1; *p* = 0.207; LR Chi-square = 7.799; df = 5; *p* = 0.576).

### 3.3. Parasitoid Host Ranges

**Figure 3:**
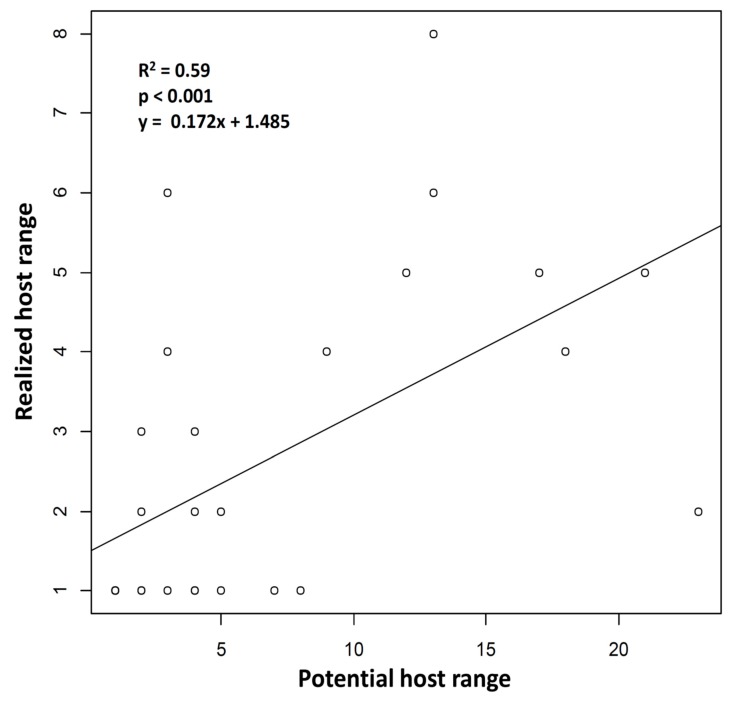
Correlation between potential host ranges and realized host ranges of the parasitoid species identified in this study. The two types of host ranges are significantly correlated (Spearman’s correlation test).

Among the 29 parasitoid species, 14 were found only on one aphid host (Figure 3, Table 2). The highest number of observed host species for a parasitoid species was recorded for *A. matricariae* with 8 host aphids. The average host range was 2.59 + 1.97. The realized host ranges were significantly correlated with the potential host ranges (Spearman’s correlation test, R^2^ = 0.593; S = 1488.1; *p* = < 0.001; Figure 3). Among the most generalist parasitoid species identified, the majority were found predominantly on one aphid species (named main host in Table 2), while other hosts were only marginally parasitized. Lastly, 21 of the aphid–parasitoid interactions observed in the agroecosystem studied had not previously been described in the literature.

The realized host range had a significant effect on the number of months of presence in nature: parasitoids with a wider host range were found to have a longer presence in the field (Table 3). However, the potential host range did not affect the duration of the presence of parasitoids. Similarly, only the realized host range affected the number of samples we collected where at least one parasitoid was found: the more generalist parasitoids were found more frequently (Table 3). Lastly, the number of collected individuals was also significantly affected by the realized host range only (Table 3).

Three factors affected the potential host range of parasitoids collected: the sampling season, the type of habitat and the interaction between the aphid supply and the type of habitat (Table 4). Indeed, the more generalist parasitoids (in theory) are preferentially collected after harvesting and in cultivated areas.

However, we did not find any effect of aphid availability, sampling season and the type of habitat sampled on the realized host range of parasitoids: none of these factors alone significantly influenced the degree of parasitoid specialization according to their realized host range (Table 4). We only found a significant effect of the interaction between all of these three factors on the realized host range of the parasitoid species collected.

### 3.4. DNA Sequencing of the Most Generalist Parasitoid Species

**Figure 4:**
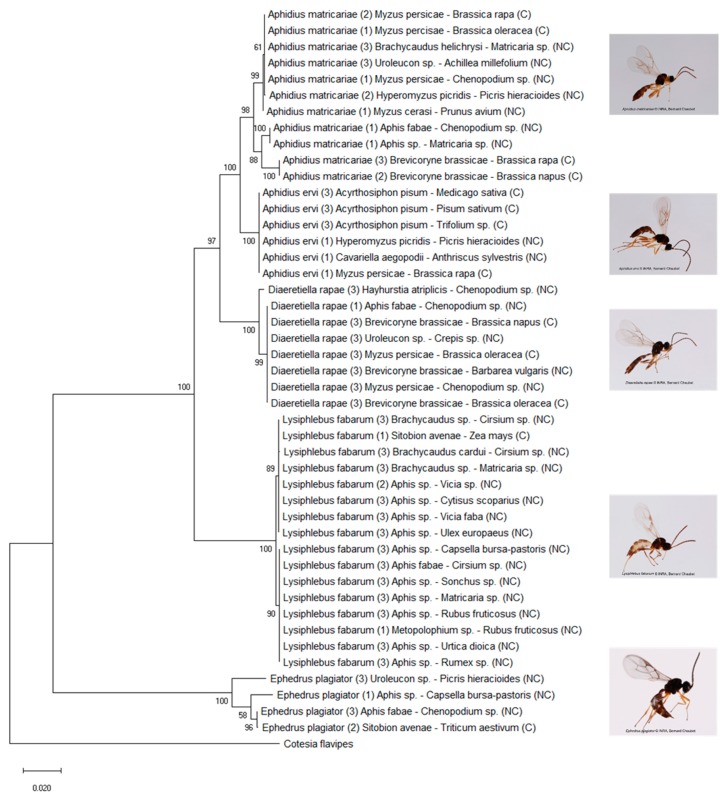
Maximum likelihood tree of the five most generalist parasitoid species based on three gene fragments (16s, COI, and LWRh). Bootstrap values are given for each branch. Scaling is expressed in the proportion of substituted bases per site. Names are constituted in the following order: parasitoid species, number of specimens sequenced between brackets, aphid host, plant host, type of habitats between brackets (C: cultivated, NC: uncultivated).

The five most abundant generalist species (with a potential host range > 10 in our study, Table 2, Figure 3) were found by us to be *A. ervi*, *A. matricariae*, *D. rapae*, *E. plagiator,* and *L. fabarum*. They were also the only ones that parasitized aphids both in cultivated and in uncultivated habitats. The multilocus maximum likelihood tree revealed clades related to the aphid host or plant family in most of these generalist species (Figure 4).

In *A. matricariae*, individuals were structured into three units: a clade including individuals from *B. brassicae* in the cultivated area, a clade including individuals from *Aphis* spp. in the uncultivated area, and a clade including individuals from five different aphid taxa and from both habitats. *Diaeretiella rapae* was separated into two groups: a group emerging from *H. atriplicis* and a group including parasitoids from other aphid hosts. In *E. plagiator*, a group including individuals emerging from *Sitobion avenae* and *Aphis fabae* was separated from the other *E. plagiator*. *Lysiphlebus fabarum* was split into two groups. The first group consisted of individuals emerging from *S. avenae*, *Brachycaudus* sp., and the *Aphis* species collected on Fabaceae in the uncultivated area. Individuals clustered in the second group exploited species of *Aphis* found on various plant families (except Fabaceae) and *Metopolophium* sp. on Rosaceae. Lastly, in *A. ervi* no structure emerged.

According to the phylogenetic analysis, we separated the following parasitoid species in distinct putative species and calculated the new realized host range for each of these following groups:

- *Aphidius matricariae*: group 1 with a realized host range of 5, group 2 with a realized host range of 2, group 3 with a realized host range of 1
- *Diaeretiella rapae*: group 1 with a realized host range of 4, group 2 with a realized host range of 1
- *Ephedrus plagiator*: group 1 with a realized host range of 2, group 2 with a realized host range of 2
- *Lysiphlebus fabarum*: group 1 with a realized host range of 4, group 2 with a realized host range of 3

The new observed realized host ranges were still significantly correlated with the potential host ranges (Spearman correlation test, R^2^ = 0.437; S = 3682.8; *p* < 0.001), although the correlation coefficient R^2^ was lower. The number of months of presence remained significantly influenced by the realized host range (GLM, LR Chi-square = 7.656; df = 1; *p* = 0.006). Similarly, the number of samples where at least one parasitoid species was found still remained significantly influenced by the realized host range (GLM, LR Chi-square = 70.288; df = 1; *p* < 0.001) as well as the number of parasitoids collected (GLM, LR Chi-square = 10.077; df = 1; *p* = 0.002).

Unlike the results obtained before the DNA-based redefining of parasitoid species, we found a significant effect of the sampling season and interaction between sampling season and habitat type on the realized host range of collected parasitoid species (Table 5).

## 4. Discussion

Studying aphid–parasitoid interactions at a local scale, this allowed us to identify the host resources actually exploited by parasitoids at this spatial scale, whether they are considered as specialists or generalists at a global scale. Our results clearly show that the great majority of Aphidiinae species used fewer host species than expected according to data as published in the scientific literature, parasitizing mainly a limited number of hosts even when other potential hosts are available in the environment. Moreover, our molecular data showed clades related to the aphid or the plant host in the species with the wider host ranges, suggesting a local host–plant specialization in the most generalist species.

*Observed host ranges*. The biodiversity observed in the chosen area was high, as we sampled about 1/10 of the aphid species described in Europe (47/404; see Supplementary Material 1 for a list of aphid species) and 1/4 of the Aphidiinae species (29/120) described in France [66]. Several highly generalist species, like *D. rapae*, *P. volucre*, or *E. plagiator*, were present in the studied area.

The almost exhaustive inventory of aphids in the studied agroecosystem made possible the fine characterization of resource availability for parasitoids over a complete season (from April to November). More importantly, this allows one to characterize the host range of parasitoids at a local scale. Except for three species known as monophagous (*A. eadyi*, *P. barbatum*, *P. uroleucon*) [36], all sampled parasitoids actually exploited a narrower host range than expected, regarding these available resources. Consequently, ten more species behaved as strictly specialist species (*A. absinthii*, *A. rosae*, *A. urticae, E. nacheri*, *E. niger*, *L. confusus*, *L. testaceipes*, *M. crepidis*, *P. yomenae, T. auctus*). Of the remaining species, very few parasitized more than three aphid taxa. Nevertheless, the observed host ranges, although narrower than described in the literature, were still consistent with expectations based on potential host ranges: the most theoretical generalist species remain the most actual generalist ones at the local scale.

Previous studies have shown that parasitoid host range might not be consistent across the entire area of geographical distribution of a species [67,68,69]. Indeed, all hosts belonging to the theoretical host range of a parasitoid are not equally preferred or suitable locally [38,40,70,71,72]. At a local scale, we observed the same pattern with potential hosts being neglected and others being predominantly exploited. The most generalist species were mainly found on one aphid species, with fewer interactions with the other host species. The only exception was *A. matricariae* in which this pattern is more balanced. In fact, none of the species observed in our study were true generalist species. The species exhibiting the larger host ranges could rather be classified as facultative specialists [73] that is "species in which individuals are adapted to exploit a single food type (here a single host species) but will exploit other niches (hosts) either opportunistically or when primary food (host) is in short supply." These species differ from obligate specialists because they perform better on their preferred host (following the preference/performance hypothesis [74]), but are able to use a range of less preferred host species. In our study, we found several previously undescribed interactions between aphids and parasitoids, the correct identification of which was confirmed by careful re-examination of both aphids and parasitoids. Moreover, some of these unusual associations were also detected in living aphids by molecular methods from the same sampling campaign (*A. ervi* on *C. aegopodii* and *A. matricariae* on *Hyperomyzus picridis* and *Uroleucon* sp., [75]). Such new or rare interactions are often attributed to erroneous identifications [76], and we cannot fully exclude that this could be partly the case in our study. Aphid hosts, at the mummy stages in particular, can also be misidentified leading to unusual aphid–parasitoid interactions, especially in aphid colonies with mixed species. Nevertheless, several morphological criteria available on aphid mummies (e.g., length of cornicles) limit the probability of misidentification, in colonies with mixed aphid species. Lastly, unusual associations can also be linked to the ability of facultative specialists to parasitize opportunistically and obtain a few offspring on less suitable hosts [38].

*DNA-based differentiation*. The higher level of specialization than expected in aphidiine parasitoids at a local scale is reinforced by the detection of genetic subgroups in the ML tree between populations of generalist species at this spatial scale, ranging from the absence of any clearly identifiable structure to the possible existence of cryptic species. The clades revealed by the ML were mostly linked to aphid taxa, host plant or both. Only one generalist species did not exhibit any identifiable genetic structure: *A. ervi*. This species exploits mainly aphids attacking Fabaceae crops, including *Acyrthosiphon pisum* [34,77], and could be considered as a true generalist in the cultivated area. *Aphidius ervi* was not well represented in the uncultivated areas with only rare events of parasitism detected on non-pest aphids, as demonstrated in previous studies [5,78]. A more solid conclusion about the absence of genetic structuring in *A. ervi* would require sampling with a particular focus on finding specimens on a wider range of aphid hosts, as only four aphid species were found parasitized by *A. ervi* in our study. Moreover, as only three parasitoids were sequenced per plant–aphid association, the sequencing of a higher number of parasitoids would be required to draw a more definitive conclusion on the following pattern found.

Samples of *A. matricariae* were separated into three groups according to the aphid host species. We observed two clades specific to a single aphid taxon and a clade gathering individuals collected on several aphids, both in the cultivated and uncultivated areas. Such a pattern has already been found [5], but at a larger geographical scale, *A. matricariae* consisted of several phylogenetic lineages (paraphyletic groups) based on the analysis of samples originating from various aphid hosts (some of them shared with this study) and various geographic areas (France, U.K., Chile). Our study confirms that *A. matricariae* is probably a cryptic species complex and demonstrates that this pattern remains at the local scale. 

Concerning *D. rapae*, individuals collected on *Hayhurstia atriplicis* were separated from those derived from other hosts, on which no difference in DNA sequences was found. This confirms the existence of a cryptic species as recently proposed [39] based on laboratory experiments (host switching, reproductive incompatibility) and molecular analyses [5,39]. Here, we confirm that both taxa co-exist locally.

In *L. fabarum*, the clades found were related to the plant of collection and we observed two clades exploiting two or three species of aphids in the uncultivated area but very few pest aphid taxa. Other studies have also demonstrated that *L. fabarum* consisted of several phylogenetic lineages [5,41,79]. A recent study showed that a morphometric measure of forewing shape is helpful to delineate cryptic species in *L. fabarum* [80]. As for *A. matricariae*, the present study has demonstrated the existence of two phylogenetic clades at a small local scale. 

Lastly, *E. plagiator* also exhibited a high intraspecific genetic variability linked to the exploited aphid host and plant [5,34,42,52]. Observations were insufficient to conclude on the importance of which factors induce population structure in *E. plagiator*, but the general pattern of a local genetic structure according to the aphid host species seems to apply to *E. plagiator* as well.

*Factors related to local specialization*. Despite the high level of host specialization locally observed, our results showed that parasitoids exploiting the wider range of aphid hosts were detected during a longer period in the agroecosystem and tended to be more abundant. This trend persisted even when considering the host ranges in the intraspecific clades (i.e., after splitting the most generalist parasitoid species according to the phylogenetic clade revealed by the ML tree and calculating the new host range of each clade). Despite these advantages, we did not observe a predominance of generalist species at the local scale. This suggests that other factors could counter-balance the benefit of being able to exploit various host species. Ecological specialization could arise from the adaptation to ecological characteristics of aphid hosts [26,75,81] or host plants such as their abundance, distribution and physiology [74,82,83]. The clades detected in the most generalist species were associated in most cases with aphid and/or plant species. This suggests that specialization relies mainly on behavioral and physiological abilities to detect and develop on a particular host. For example, exploiting the aphid *H. atriplicis*, which develops in pseudo-galls on *Chenopodium* sp. leaves implies that the emerging parasitoids are able to make holes in these pseudo-galls [39]. Such behavioral adaptations could explain, at least partially, the divergence in populations of *D. rapae* exploiting this aphid or *B. brassicae* as a major host, leading to speciation.

Moreover, specialization could result in a low level of competition between species [6] for the host resources. When considering the main host used by each parasitoid species, we observed a limited overlap between parasitoid species supporting the view that specialization promotes efficient resource sharing. However, two species in the cultivated area, *A. pisum* and *S. avenae*, were the main hosts of several parasitoids, specialist as well as a generalist (e.g., *A. eadyi* and *A. ervi* on *A.pisum* or *A. rhopalosiphi* and *A. avenae* on *S. avenae*). However, these species could avoid competition by temporal host sharing [84]. Moreover, aphid abundances were high in the studied environment, probably limiting direct competition.

We found no general effect of the type of habitat and the aphid abundance on the realized host range. The hypothesis that generalist species would be more abundant in the unstable, cultivated area was not verified here, although more parasitized aphids were found in this habitat. Furthermore, we did not find any evidence that the more generalist species exploit other hosts after harvesting (i.e., after their main host disappeared). Finally, because aphid abundance has no effect on host exploitation by parasitoids, we can expect that the variation of this abundance between years would weakly impact the observed host range. However, this remains to be checked by additional sampling. 

Delimiting the host ranges of parasitoids and understanding the factors that shape them has often been undertaken by compiling data originating from a large geographical area and several years. This approach allows the development of hypotheses about the selective forces driving the level of host specialization in parasitoid species. For instance, Gagic et al. [76] recently identified some important host traits associated with aphid–parasitoid specificity in a 13-year survey, in nine European countries. It should also be useful to consider host–parasitoid interactions at more local and short terms scales, as recommended by Trojelsgaard and Olesen [85] for the study of ecological networks. By doing so, we confirmed that Aphidiine species are more highly specialized than expected. All the species we found can be classified as obligate specialists and facultative specialists [69], or monophagous and oligophagous [6]. Specialization appears to arise at infraspecific levels in many of the latter. The factors identified (e.g., density and concealment of aphid colony, mobility of aphid species) by Gagic et al. [76] should be re-examined at this local scale to check the consistency of host range diversity between large and narrow spatial and temporal scales. Due to the high dispersal abilities of aphids and parasitoids [86], assessing the realized host ranges at nested spatial scales would fill the gap between very local and continental scale studies. 

## 5. Conclusions

Our study has clearly revealed that aphid parasitoids hitherto considered as generalists behave as specialists at a local scale. While more effort still needs to be made in relation to the DNA-based side of this study by providing a higher number of specimens sequenced as well as additional years of sampling data, our results confirm that most generalist species are actually composed of specialist clades. In addition to confirming that true generalist aphid parasitoids are actually scarce in nature, we demonstrated that this pattern (usually shown at very large geographical and time scales) persists at the agroecosystem scale. It is of great importance to consider such local specialization in order to enhance pest regulation within agroecosystems by providing the right resources in terms of parasitoid presence and hence impact.

## Figures and Tables

**Table 1:** Primer pairs used in this study to amplify and to sequence the DNA of parasitoid species.

Primer	Gene Amplified	PCR Product	Sequences	References
LCO1490	COI	620 bp	5’-GGTCAACAAATCATAAAGATATTGG-3’	[53]
HCO2198	COI	620 bp	5’-TAAACTTCAGGGTGACCAAAAAATCA-3’	[53]
16S-F	16S	380 bp	5’-CGC CGT TTT ATC AAA AAC ATG T-3’	[54]
16S-R	16S	380 bp	5’-TTA CGC TGT TAT CCC TAA-3’	[55]
LWRhF	LWRh	650 bp	5’-AAT TGC TAT TAY GAR CAN TGG GT-3’	[56]
LWRhR	LWRh	650 bp	5’-ATA TGG AGT CCA NGC CAT RAA CCA-3’	[56]

**Table 2:** List of Aphidiinae species present in the agroecosystem with their potential host range size (based on the literature) and their realized host ranges based on the field sampling.

Parasitoids Species	Hosts Not Described in the Literature	Main Host (Number of Samples)	Presence in the Agroecosystem	Month(s) of Presence	Number of Samples	Number of Individuals	Potential Host Range	Realized Host Range
*Adialytus salicaphis*		*Chaitophorus leucomelas* (2)	June, July	2	2	36	4	2
*Aphidius absinthii*		*Macrosiphoniella* sp. (1)	June	1	1	1	2	1
*Aphidius avenae*	*Aphis fabae*	*Sitobion avenae* (12)	April to July	4	16	50	9	4
*Aphidius eadyi*		*Acyrthosiphon pisum* (1)	Avril	1	1	1	1	1
*Aphidius ervi*	*Cavariella aegopodii and Hyperomyzus picridis*	*Acyrthosiphon pisum* (8)	April to June, September, October	5	12	40	17	5
*Aphidius funebris*	*Uroleucon* sp. (*Uromelan* sp.)	*Uroleucon* sp. (10)	June, July, September, October	4	15	36	2	3
*Aphidius matricariae*	*Hyperomyzus picridis and Uroleucon* sp.	*Myzus persicae* (3)	May to July, October	4	11	23	13	8
*Aphidius rhopalosiphi*		*Sitobion avenae* (2)	April, May	2	3	5	2	2
*Aphidius rosae*		*Macrosiphum funestum* (1)	June	1	1	1	2	1
*Aphidius salicis*		*Cavariella aegopodii* (1)	June	1	2	22	5	2
*Aphidius sonchi*	*Aphis fabae, Cavariella pastinaceae and Uroleucon* sp.	*Hyperomyzus picridis* (2)	Mai, June	2	6	92	3	6
*Aphidius urticae*		*Acyrthosiphon pisum* (1)	April	1	1	1	8	1
*Binodoxys acalephae*	*Cavariella aegopodii, Macrosiphoniella* sp. *and Uroleucon* sp.	*Aphis* sp. (2)	June, July	2	5	20	3	4
*Binodoxys angelicae*	*Cavariella theobaldii and Hyadaphis foeniculi*	*Aphis* sp. (7)	May at July, September	4	18	75	12	5
*Binodoxys centaureae*	*Aphis* sp *and Uroleucon* sp. (*Uromelan* sp.)	*Uroleucon* sp. (4)	June, July	2	7	36	4	3
*Diaeretiella rapae*		*Brevicoryne brassicae* (9)	May, June, September, October	4	19	320	21	5
*Ephedrus nacheri*		*Hayhurstia atriplicis* (3)	September, October	2	3	6	3	1
*Ephedrus niger*		*Uroleucon* sp. (1)	July	1	1	2	4	1
*Ephedrus plagiator*	*Uroleucon* sp.	*Uroleucon* sp. (6)	June, October, November	3	10	24	18	4
*Lysiphlebus confusus*		*Aphis* sp. (1)	June	1	1	5	7	1
*Lysiphlebus fabarum*	*Metopolophium* sp.	*Aphis* sp. (14)	June, July, October	3	23	696	13	6
*Lysiphlebus testraceipes*	*Macrosiphum* sp.	*Macrosiphum* sp. (1)	June	1	1	19	5	1
*Monoctonus crepidis*	*Uroleucon* sp.	*Uroleucon* sp. (2)	June	1	2	19	1	1
*Praon barbatum*		*Acyrthosiphon pisum* (2)	June, September	2	2	2	1	1
*Praon* sp.		*Uroleucon* sp. (1)	July	1	1	7	NA	1
*Praon uroleucon*		*Uroleucon* sp. (2)	June, July	2	2	2	1	1
*Praon volucre*		*Uroleucon* sp. (2)	June, July	2	3	9	23	2
*Praon yomenae*		*Uroleucon* sp. (2)	June	1	2	4	4	1
*Trioxys auctus*	*Sitobion avenae*	*Sitobion avenae* (1)	July	1	1	1	1	1

**Table 3:** Effect of host range size (realized and potential) of the parasitoid collected on the number of (left) months of presence in the field, (middle) samples collected where at least one parasitoid was found, (right) parasitoid individuals collected in the field (generalized linear model—GLM).

Factors Tested	Months of Presence			Samples Collected			Parasitoids Collected		
	LR Chi-Square	Df	*p*-Value	LR Chi-Square	Df	*p*-Value	LR Chi-Square	Df	*p*-Value
Realized host range	5.168	1	0.023	26.357	1	<0.001	30.039	1	<0.001
Potential host range	0.893	1	0.345	0.765	1	0.382	0.444	1	0.505
Potential host range:Realized host range	0.031	1	0.86	0.083	1	0.773	0.2	1	0.655

**Table 4:** Effect of aphid abundance, sampling season and type of habitat on (left) the realized host range size of parasitoids collected, (right) the potential host range size of parasitoids collected (GLM).

Factors Tested	Realized Host Range			Potential Host Range		
	LR Chi-Square	Df	*p*-Value	LR Chi-Square	Df	*p*-Value
Aphid abundance	1.35	1	0.245	0.024	1	0.876
Sampling season (i.e., before/after harvest)	2.47	1	0.116	4.086	1	0.043
Type of habitat (i.e., uncultivated/cultivated)	0.063	1	0.802	5.952	1	0.015
Aphid abundance:season	0.175	1	0.676	1.275	1	0.259
Aphid abundance:type of habitat	3.165	1	0.075	5.86	1	0.015
Season:habitat	1.054	1	0.305	2.038	1	0.153
Aphid abundance:season:habitat	5.588	1	0.018	0.616	1	0.433

**Table 5:** Effect of aphid abundance, sampling season and type of habitat on the realized host range size of parasitoids collected once five parasitoid species were separated in groups according to the clades revealed by the ML tree (GLM).

Factors Tested	LR Chi-Square	Df	*p*-Value
Aphid abundance	0.298	1	0.585
Sampling season (i.e., before/after harvest)	15.727	1	<0.001
Type of habitat (i.e., uncultivated/cultivated)	0.33	1	0.566
Aphid abundance:season	0.047	1	0.828
Aphid abundance:type of habitat	0.513	1	0.4740
Season:habitat	3.999	1	0.046
Aphid abundance:season:habitat	1.615	1	0.204

## Supplementary Materials

The following are available online at https://www.mdpi.com/2075-4450/11/1/6/s1. Supplementary material 1: List of the aphid species sampled in the study; Supplementary material 2: spreadsheets of the sampling data.
