# Supplementary material for: “Generalist” Aphid Parasitoids Behave as Specialists at the Agroecosystem Scale
Source: Insects. 2019 Dec 19;11(1):6. doi: 10.3390/insects11010006 (PMC7023390; doi:10.3390/insects11010006)
Supplement: Supplementary file 1 [file insects-11-00006-s001.pdf]

Supplementary Material. List of aphid species sampled in the study where at least one parasitoid was detected

Acyrthosiphon pisum
Aphis fabae
Aphis farinosa
Aphis sp.
Aulacorthum solani
Brachycaudus cardui
Brachycaudus helichrysi
Brachycaudus sp.
Brevicoryne brassicae
Cavariella aegopodii
Cavariella pastinaceae
Cavariella sp.
Cavariella theobaldii
Chaitophorus leucomelas
Chaitophorus sp.
Hayhurstia atriplicis
Hyadaphis foeniculi
Hyperomyzus lactucae
Hyperomyzus picridis
Hyperomyzus sp.
Macrosiphoniella sp.
Macrosiphum funestum
Macrosiphum sp.
Metopolophium dirhodum
Metopolophium sp.
Microlophium carnosum
Myzus cerasi
Myzus persicae
Sitobion avenae
Uroleucon sonchi
Uroleucon sp.
Uroleucon sp. (*Uromelan* sp.)

Supplementary Material. List of aphid species sampled in the study without any parasitoid detected

Amphorophora rubi
Aphis cytisorum
Aulacorthum solani
Betulaphis quadrituberculata
Callipterinella calliptera
Corylobium avellanae
Elatobium abietinum
Glyphina betulae
Hyadaphis foenicul
Macrosiphoniella millefolii
Macrosiphum euphorbiae
Megoura viciae
Thelaxes dryophila
Utamphorophora humbolti
